# Determination of seroprevalence and kinetics of humoral response using mpox virus A29 protein

**DOI:** 10.1038/s43856-023-00403-9

**Published:** 2023-11-22

**Authors:** Jian-Piao Cai, Wing-Ming Chu, Anthony Raymond Tam, Kun Wang, Yuting Han, Lin-Lei Chen, Xiaojuan Zhang, Charlotte Yee-Ki Choi, Vincent Chi-Chung Cheng, Kwok-Hung Chan, Zhiwei Chen, Ivan Fan-Ngai Hung, Carol Ho-Yan Fong, Kelvin Kai-Wang To

**Affiliations:** 1 State Key Laboratory for Emerging Infectious Diseases, Carol Yu Centre for Infection, Department of Microbiology, School of Clinical Medicine, Li Ka Shing Faculty of Medicine, The University of Hong Kong, Pokfulam, Hong Kong Special Administrative Region China; 2 Infectious Diseases Division, Department of Medicine, Queen Mary Hospital, Pokfulam, Hong Kong Special Administrative Region China; 3 Department of Microbiology, Queen Mary Hospital, Pokfulam, Hong Kong Special Administrative Region China; 4 Centre for Virology, Vaccinology and Therapeutics, Hong Kong Science and Technology Park, Hong Kong Special Administrative Region, China; 5 AIDS Institute, Li Ka Shing Faculty of Medicine, The University of Hong Kong, Pokfulam, Hong Kong Special Administrative Region China; 6 Department of Medicine, School of Clinical Medicine, Li Ka Shing Faculty of Medicine, The University of Hong Kong, Pokfulam, Hong Kong Special Administrative Region China; 7 Department of Infectious Disease and Microbiology, The University of Hong Kong-Shenzhen Hospital, Shenzhen, Guangdong, China

**Keywords:** SARS-CoV-2, Epidemiology

## Abstract

**Background:**

Mpox virus (MPXV), previously known as monkeypox virus, has spread globally in 2022. An accurate and convenient antibody test is essential for the determination of seroprevalence and for studying immune response after natural infection or vaccination. Most seroprevalence or vaccine studies used either live MPXV (or vaccinia virus [VACV]) or inactivated MPXV (or VACV) culture lysate for serological assays, but MPXV culture can only be performed in biosafety level 3 (BSL-3) facilities. Here, we developed and evaluated an enzyme immunoassay (EIA) based on the MPXV A29 surface envelope protein.

**Methods:**

We compared the specificity of the MPXV A29, VACV A27, and VACV lysate EIA using serum specimens collected prior to the global spread of MPXV. Next, we performed these EIAs for serum specimens collected from two mpox patients and an MVA-BN vaccine recipient. We also assessed the kinetics of plasmblast and MPXV A29-specific B-cell response.

**Results:**

Using sera collected from different age groups in Hong Kong, we found that most individuals, including those born before 1981 who have received the smallpox vaccine, tested negative using the MPXV A29 protein. MPXV A29-specific antibody could be detected in the serum of mpox patients and an MVA-BN recipient. In a mpox patient, the frequency of plasmablast and MPXV A29-specific B cell peaked on day 8 post-symptom onset and gradually decreased. Finally, we demonstrated that antibodies against the A29 protein can be used for immunofluorescence staining of MPXV-infected cells.

**Conclusions:**

MPXV A29 protein is suitable for studying the immune response against MPXV infection.

## Abstract (web summary)

Cai et al. develop and evaluate mpox A29 surface envelop protein-based enzyme immunoassay to analyze seroprevalence and B cell responses after natural mpox viral infection or vaccination. The assay can readily detect mpox A29-specific B cells and antibody in the serum of mpox patients and MVA-BN vaccine recipients.

## Plain language summary

Since early 2022, mpox (monkeypox) has been reported in many countries where the disease is not regularly found to occur. The aim of the study was to develop and evaluate the performance of laboratory assays based on the mpox virus surface protein, named A29. We found our assays could accurately distinguish naturally infected cases from smallpox vaccine recipients as well as those who were neither infected nor vaccinated. Our assays provide a useful tool for studying the host immune response to mpox virus.

## Introduction

Mpox virus (MPXV), previously known as monkeypox virus, is a zoonotic virus that was geographically restricted to Africa before 2022. Clades I and IIa were related to the MPXV infection in Central Africa and West Africa, respectively. However, clade IIb, first identified in Nigeria in the 1970s, has spread rapidly worldwide in 2022. As a result, the World Health Organization has declared the MPXV outbreak as a public health emergency of international concern on 23 July 2022^1^.

Antibody testing for MPXV is essential for vaccine trials and seroprevalence studies. The gold standard for vaccine studies is the neutralizing antibody (nAb) response^2^. However, the live virus nAb test is labor-intensive and can only be performed in biosafety level 3 (BSL-3) facilities. Enzyme immunoassay (EIA) based on MPXV cell culture lysate has been reported^3^, but the large volume of live MPXV required for the production of MPXV lysate poses potential safety concerns. Previous serosurveillance studies for MPXV used EIA with vaccinia virus (VACV) coated plates^4^, but these VACV EIA cannot differentiate between smallpox vaccination and MPXV infection. By peptide library screening, Dubois et al. have identified MPXV B21R protein that could differentiate individuals immunized with the vaccinia virus from patients who had MPXV infection with EIA^5^. However, this test was evaluated in a small number of individuals who were previously immunized with the vaccinia virus vaccine. There was also an attempt to differentiate between mpox patients from vaccinia vaccine recipients using differential western blot analysis^3^, but the results from this assay can be difficult to interpret.

Roumillat et al. identified a monoclonal Ab (mAb 69-126-3-7) that could bind but not neutralize MPXV^6^. Hughes et al. subsequently showed that mAb 69-126-3-7 could bind specifically to the MPXV A29 protein but not the ortholog A27 of VACV^7^, and the specificity was related to the difference at amino acid position 27 (lysine in A27 and asparagine in A29 protein). Gilchuk et al. found that VACV A27 protein is a target of nAb^8^, and is also a T-cell epitope^9^.

Here, we demonstrated that antibodies against MPXV A29 could not be detected for most non-infected individuals, including those who had prior smallpox vaccine before 1980, but could be detected in mpox-infected individuals and an modified vaccinia Ankara–Bavarian (MVA-BN) vaccine recipient with western blot. Furthermore, MPXV A29 protein could be used as the target for assessing B-cell kinetics.

## Methods

### Patients and study approval

Anonymized archived serum samples from the clinical biochemistry laboratory of Queen Mary Hospital in Hong Kong were used for serological study as we described previously^10^. The archived specimens encompassed all age groups from 0–9 to ≥80 years old. A total of 443 serum specimens collected between January and April 2022 were randomly selected and tested. Written informed consent was waived since archived anonymized specimens were used.

The clinical presentation, laboratory findings, and viral loads of mpox patient 1 were described previously^11^. Serial saliva and blood specimens were collected for viral culture and antibody/B-cell assays, respectively. Written informed consent was obtained. The serum from the vaccine recipient was collected 8 months after the second dose of MVA–BN (JYNNEOS®, Bavarian Nordic), which is a live vaccine produced from the strain MVA-BN.

This study was approved by the Institutional Review Board of the University of Hong Kong/Hospital Authority Hong Kong West Cluster (UW 13-265 and UW 18-141).

### Amino acid alignment of VACV A27, MPXV A29 and variola virus (VARV) A30

The amino acid sequence of VACV A27, MPXV A29, and VARV A30 were obtained from GenBank (accession number: YP_233032.1, AAL40597.1, and NP 042178.1). Amino acid alignment of MPXV A29, VACV A27, and VARV A30 was performed using PRALINE multiple sequence alignment (https://www.ibi.vu.nl/programs/pralinewww/).

### Expression and purification of MPXV A29 and VACV A27 proteins

A29L of MPXV with the reference sequence Zaire-96-I-16 (GenBank AF380138.1) and A27 of VACV with the reference sequence “Western Reserve” (GenBank NC_006998.1) was codon-optimized and cloned into pET-28b(+) expression vector with an N-terminal 6x-His tag for purification (Sangon Biotech). The expression and purification procedure were performed as we described previously with modifications^12^. Briefly, the MPXV A29L or VACV A27 plasmid was transformed to *Escherichia coli* strain BL21-Gold (DE3) (Agilent, Catalog no. 230132) for protein expression. A single cloned BL21-Gold (DE3) was selected and inoculated into a 10 mL LB broth (Thermo Fisher Scientific, Catalog no. 12780029) and incubated at 37 °C. The bacterial culture was shaken at 250 rpm until reaching the exponential phase as indicated with OD600 of 0.6–0.8. Isopropyl β-D-1-thiogalactopyranoside (IPTG) was added to the bacterial culture with a final concentration of 0.1 mM in the medium. The bacterial culture was then incubated at 16 °C with shaking at 250 rpm overnight. The culture was then centrifuged and the cell pellet was dissolved with 1x phosphate-buffered saline (PBS), pH 7.4, and followed by sonication. The MPXV A29 or VACV A27 protein in the supernatant was purified by the Ni-NTA purification system (Thermo Fisher Scientific, Catalog no. R90110), followed by size exclusion chromatography (Bio-Rad, USA ENrich™ SEC 650 10 × 300 Column, Catalog no. 7801650) and buffer exchanged into 1x PBS pH 7.4. The concentration of MPXV A29 and VACV A27 proteins was determined using the Bradford Assay Kit (Bio-Rad, Catalog no. 5000002), and the purity was verified using sodium dodecyl sulfate-polyacrylamide gel electrophoresis (SDS-PAGE). The expression of each protein was confirmed with western blot (Supplementary Fig. S5).

### Biotinylation of MPXV A29 and VACV A27 proteins

Purified MPXV A29 and VACV A27 proteins were biotinylated as we described previously with modifications^13^. Purified MPXV A29 and VACV A27 proteins were diluted in PBS to 2 mg/mL and dispensed into glass tubes on ice. A total of 2.2 mg of EZ-link^TM^ Sulfo-NHS-Biotin (Thermo Fisher Scientific, Catalog no. 21217) was dissolved in 0.5 mL sterile H_2_O after equilibrating to room temperature. Then, 6 µL of biotin was added to 0.2 mL of diluted MPXV A29 or VACV A27 proteins and was kept on ice for 2 h. Biotinylated MPXV A29 or VACV A27 proteins were dialyzed using Slide-A-Lyzer^TM^ Dialysis Cassettes 10 K MWCO (Thermo Fisher Scientific, Catalog no. 66810) to remove unbound biotin. The dialyzed proteins were centrifuged at 12,000 rpm for 10 min under 4 °C to eliminate denatured protein. The proteins were stored with 50% glycerol under –80 °C. The quantity of biotinylated proteins was determined using the Bradford assay.

### Expression and purification of *Talaromyces marneffei* recombinant MP1P protein

The *T. marneffei* recombinant MP1P protein was expressed using the *Pichia pastoris* expression system as we described previously^14^. Briefly, the *T. marneffei* Mp1p gene was cloned into the *P. pastoris* expression vector pPIC9K (Invitrogen, Carlsbad, CA), and was transformed into the *P. pastoris*. The recombinant protein expressed from the Mp1p transformed *P. pastoris* was then purified using the Ni-NTA purification system (Thermo Fisher Scientific, Catalog no. R90110), followed by size exclusion chromatography (Bio-Rad, USA ENrich™ SEC 650 10 ×300 Column, Catalog 7801650) and buffer exchanged into 1x PBS pH 7.4. The purity was verified using SDS-PAGE.

### VACV inactivation and purification

VACV Tian Tan strain (GenBank accession number: AF095689.1) was cultured in VeroE6 cells for 48 h. To inactivate the virus, the viral culture supernatant was mixed with 0.03% (v/v) formalin and was incubated at 4 °C for 7 days with shaking. The efficiency of inactivation was confirmed by plaque assay^15^. The inactivated virus was then purified and concentrated by sucrose gradient ultra-centrifugation at 28,000 rpm for 2 h^16^. The concentration of inactivated virus was quantified using the Bradford Assay Kit, aliquot and stored at −80 °C until use.

### Generation of mouse mAb against MPXV A29 protein

The generation of mouse mAb against MPXV A29 protein was performed as we described previously with modifications^14^. Four to six-weeks-old female BALB/c mice (*n* = 2), were immunized subcutaneously with 20 μg of recombinant MPXV A29 protein that was pre-mixed with an equal volume of complete Freund′s adjuvant (Sigma-Aldrich, Catalog no. F5881). Following the first immunization, mice were given 20 μg of MPXV A29 protein pre-mixed with incomplete Freund′s Adjuvant (Sigma-Aldrich, Catalog no. F5506) on days 10, 20 and 30 subcutaneously. On day 40, mice were boosted with 30 μg MPXV A29 protein intravenously without adjuvant. Spleen was harvested on day 43 and the splenocyte was fused with myeloma cells to generate hybridoma cells under the selection of hypoxanthine-aminopterin-thymidine (HAT) medium. Hybridoma clones were screened for the presence of MPXV A29-specific mAbs by EIA. MPXV A29-specific mAbs were then harvested from hybridoma cell culture supernatant, and purified by filtering through 0.22 µm filters and chromatography cartridge protein A/G (Thermo Fisher Scientific, Catalog no. 89931) at room temperature (RT). Monoclonal Abs were washed with PBS (pH 7.4) eluted using 0.1 M sodium citrate (pH 3.0) and neutralized with 1 M Tris-HCl (pH 8.8). Non-specific proteins were further removed using size exclusion chromatography. The purified mAbs were then buffer exchanged into 1× PBS (pH 7.4) and the concentration was measured using the Bradford Assay Kit.

Animal experiments were performed at the University of Hong Kong and conducted in compliance with all animal protocols approved by the Committee on the Use of Live Animals in Teaching and Research of the University of Hong Kong (CULATR 22-242). BALB/c mice were originally sourced from The Center for Comparative Medicine Research, University of Hong Kong, and housed under an AAALAC International accredited program. 12:12 dark light cycle within environmentally controlled rooms was provided in an animal facility and mice were fed ad libitum with a laboratory diet manufactured by Harlan UK Ltd, UK.

### Enzyme immunoassay

96-well polystyrene high binding microplate (Corning, (Catalog no. 3690) were coated with 50 μL of His-tagged MPXV A29 or VACV A27 protein (50 ng/well) or inactivated VACV lysate (500 ng/well) in 0.05 M NaHCO_3_ (pH 9.6) overnight at 4 °C. After blocking with blocking reagent at 37 °C for 2 h, 50 μL of heat-inactivated human serum samples at 1:200 dilution or 2-fold serial dilution of mouse anti-MPXV A29 (5H9) or mouse anti-rMp1p antibodies were added to the wells and incubated at 37 °C for 1 h. Human and mouse immunoglobulin G (IgG) were detected using horseradish-peroxidase (HRP)-conjugated goat anti-human IgG (Thermo Fisher Scientific, Catalog no. A18847) or goat anti-mouse IgG antibody, respectively (Thermo Fisher Scientific, Catalog no. 31430). The reaction was developed by adding diluted 3,3’,5,5’-tetramethylbenzidine single solution (Invitrogen, Catalog no. 002023) and stopped with 0.3 N H_2_SO_4_. The OD was read at 450 and 620 nm.

The seropositive cutoff values of VACV and MPXV EIA were set as three standard derivations above the mean of 133 archived anonymous serum specimens of patients born between the years 1993–2022 (age 0–29 years).

### IgG immunoblot assay

Western blot for anti-MPXV A29 IgG or anti-VACV A27 IgG was performed according to our previously published protocol with modifications^17^. Briefly, separated recombinant MPXV A29 or VACV A27 proteins were transferred from SDS-PAGE gel to a nitrocellulose membrane (Bio-Rad, Catalog no. 1620115). The membrane was blocked with 10% skim milk (Oxoid, Thermo Fisher Scientific, Catalog LP0031) in 1x PBS containing 0.3% (v/v) Tween 20 (Invitrogen, Thermo Fisher Scientific, Catalog P1379) at 4 °C. After overnight incubation, membrane was incubated with 1:400 diluted heat-inactivated human serum or 1:4000-diluted mouse anti-His mAb (ABclonal, Catalog AE003) in blocking buffer at RT for 45 min. The membrane was then washed three times in PBS containing 0.3% Tween 20. HRP-conjugated goat anti-human IgG (Thermo Fisher Scientific, Catalog no. A18811) or goat anti-mouse IgG antibody (Thermo Fisher Scientific, Catalog no. 31430) was added and incubated for 30 min at RT. Followed by 3 washes with PBS containing 0.3% Tween 20, the membrane was developed using Advansta ECL WesternBright^TM^ Quantum Detection Kit (Advansta, Catalog K-12042-D20).

### Peripheral blood mononuclear cells (PBMCs) isolation

Whole blood samples were collected with ethylenediaminetetraacetic acid (EDTA) BD Vacutainer® and processed on the same day. Whole blood was diluted 1:1 with RPMI containing 1% fetal bovine serum (FBS) (Gibco®, Thermo Fisher Scientific, Catalog no. 16140071) and layered onto lymphoprep^TM^ (STEMCELL Technologies, Catalog no. 07851). Diluted whole blood was centrifuged at 2000 rpm for 20 min and the PBMC fraction was collected into new tubes. PBMC were washed with RPMI containing 1% FBS, and red blood cells were removed by adding red blood cell lysis buffer (Miltenyi Biotec, Catalog no. 170-080-033) and incubated at RT for 10 min. PBMC was then washed with RPMI-1640 containing 1% FBS and filtered by a 70 μm filter. PBMC were cryopreserved in a freezing medium (90% FBS containing 10% dimethylsulfoxide) at 5 × 10^6^ cells/mL and underwent controlled freezing before storage in liquid nitrogen.

### Flow cytometry

MPXV A29-specific B cells were detected using biotinylated MPXV A29 protein in combination with two different streptavidin (SA)–fluorophore conjugates^18^. Biotinylated MPXV A29 protein was either conjugated with SA-BV421 (BioLegend, Catalog no. 405226) / SA-PE/Cy7 (BioLegend, Catalog no. 405206) at a 1.2:1 mass ratio (e.g., 200 ng of biotinylated MPXV A29 protein with ~167 ng of SA; 4:1 molar ratio), or SA-APC (Biolegend, Catalog 405207)/SA-PE/Cy7 (BioLegend, Catalog no. 405206) at a 2.4:1 mass ratio (e.g., 200 ng of biotinylated MPXV A29 protein with ~83 ng of SA; 8:1 molar ratio) for 1 h at 4 °C. Cryopreserved PBMC from the mpox patient or non-infected controls were blocked with human FcR blocking reagent (Miltenyi Biotec, Catalog no. 130-059-901) and dead cells were stained with Zombie NIR™ Fixable Viability Kit (BioLegend Catalog 423106) or Zombie violet™ Fixable Viability Kit (BioLegend, Catalog no. 423114). After 30 min of incubation at 4 °C, PBMC were washed and stained with 50 μL of antigen probe master mix containing 400 ng of biotinylated MPXV A29-PE/Cy7 and 334 ng biotinylated MPXV A29-BV421 or 166 ng of biotinylated MPXV A29-APC for 1 h at 4^ o^C. PBMC were then washed and stained with Alexa Fluor® 700 anti-human CD3 antibody (Clone SK7, BioLegend, Catalog no. 344822) PE anti-human CD19 antibody (Clone HIB19, BioLegend, Catalog 302208), APC anti-human CD27 antibody (Clone O323, BioLegend, Catalog 302810), FITC anti-human CD38 antibody (Clone HB-7, BioLegend, Catalog no. 356610) at 4 °C for 30 min. PBMC was washed and fixed with 2% paraformaldehyde for 20 min. Cells were analyzed with BD Influx^TM^ and data were analyzed using FlowJo v10 (BD Bioscience). Cells were classified as plasmablast (CD3^-^CD19^+^CD27^+^CD38^+^) and MPXV A29-specific B cells (CD3^-^CD19^+^MPXV-A29PE/Cy7^+^MPXV-A29-BV421^+^ or MPXV-A29-APC^+^).

### Viral culture and immunofluorescence staining

MPXV was isolated from the saliva specimens collected from the mpox patient and cultured in our BSL-3 laboratory. Briefly, 1 × 10^5^ VeroE6 cells (ATCC; CRL-15786) were pre-seeded in shell vials (Diagnostic Hybrids) containing 1 mL minimum essential medium (MEM) (Gibco®, Thermo Fisher Scientific) with 10% FBS. Cells were incubated at 37 °C, 5% CO_2_ until reaching 90% confluence. VeroE6 cells were then inoculated with 100 μL of saliva specimen and 100 μL viral transport medium. After 1 h incubation at 37 °C, 5% CO_2_, the diluted saliva medium was removed and replenished with 1 mL of MEM medium with 1% FBS, 100 U/mL of penicillin, 100 μg of streptomycin, 100 U/mL of nystatin, and 25 mM HEPES (Gibco®, Thermo Fisher Scientific). VeroE6 cells were then incubated at 37 °C with 5% CO_2_ for 7 days. Cytopathic effects (CPE) were examined daily with an inverted light microscope. Viral cultures expressing >50% CPE were selected and further expanded in VeroE6 cells with the conditions described.

For immunofluorescence staining, infected VeroE6 cells were harvested and washed twice in 1x PBS. The cells were smeared on a super-cured glass slide and fixed with acetone at −20^ o^C for 30 min. Mouse anti-MPXV-A29 (5H9) and anti-rMp1p antibodies at 10 μg/mL were added to the cells and incubated for 1 h at 37°. VeroE6 cells were then stained with Alexa Fluor™ 594-conjugated goat anti-mouse IgG (Thermo Fisher Scientific, Catalog no. A-11005). The slides were examined with a fluorescent microscope equipped with Image-Pro Plus 4.5 software.

### Reporting summary

Further information on research design is available in the Nature Portfolio Reporting Summary linked to this article.

## Statistics and reproducibility

Statistical analysis was performed using SPSS version 26 and GraphPad Prism (Version 9.1.2.). *Z*-test was used to compare the seropositive rates between different age groups. Between-group comparisons were performed with a two-sided chi-square test. A *P*-value of <0.05 was considered statistically significant.

## Results

**Fig. 1:**
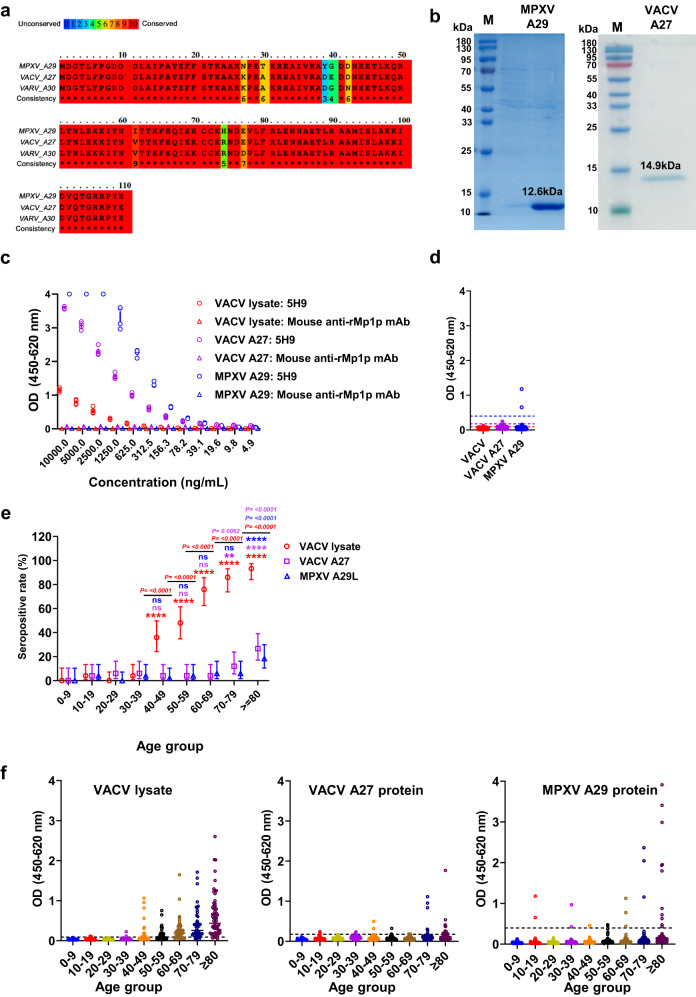
Evaluation of enzyme immunoassay for MPXV A29 protein. **a** Amino acid alignment of VACV A27, MPXV A29, and VARV A30. **b** Coomassie Blue stained SDS-PAGE showing purified recombinant MPXV A29 and VACV A27 protein expression with the expected molecular weight of 12.6 kDa and 14.9 kDa. (M) Pre-stained molecular weight protein marker, (MPXV A29) recombinant MPXV A29 protein, (VACV A27) recombinant VACV A27 protein. The original SDS-PAGE images are shown in Supplementary Fig. S6a. **c** MPXV A29 protein (blue), VACV A27 protein (violet), and VACV lysate (red) EIA with serial dilution of mouse anti-MPXV A29 mAb (5H9) and mouse anti-rMp1p mAb. The error bar represents the standard derivation of the mean from 4 technical replicates. **d** The EIA cutoff values of VACV lysate (red) MPXV A29 (blue) and VACV A27 (violet) proteins were set as three standard derivations above the mean of the 133 archived anonymous serum specimens of patients born between the years 1993–2022 (age 0–29). **e** VACV lysate (red), VACV A27 protein (violet) and MPXV A29 protein (blue) EIA seropositive rate of individual age groups from 0–9 (*n* = 33), 10–19 (*n* = 50), 20–19 (*n* = 50), 30–39 (*n* = 50), 40–49 (*n* = 50), 50–59 (*n* = 50), 60–69 (*n* = 50), 70–79 (*n* = 50), ≥80 (*n* = 60). The symbol indicates the seropositive rate (%) and the error bar indicates 95% confidence interval. A two-tailed, chi-square test was used for statistical analysis. The *P*-values represent the comparison between each age group and the 0–39 year-old age group. **f** VACV lysate, VACV A27 protein, and MPXV A29 protein EIA from sera of individuals from different age groups.

We aligned the amino acid sequences of the VACV A27 protein, MPXV A29 protein, and VARV A30 protein. The amino acid identity was 94.5% between VACV A27 and MPXV A29, 93.6% between VARV A30 and MPXV A29, and 97.3% between VARV A30 and VACV A27 (Fig. 1a). We hypothesized that MPXV A29 protein can be used for detection of MPXV-specific antibody response. We cloned and expressed the MPXV A29 protein (Fig. 1b), and generated a monoclonal antibody (mAb) against A29 protein (5H9) using mouse hybridoma. Using 5H9 mAb as a positive control and mAb against recombinant *T. marneffei* rMp1p protein as a negative control, we demonstrated that the MPXV A29 protein had a stronger signal than that of VACV lysate and VACV A27 protein (Fig. 1c).

To establish the cutoff of MPXV A29 protein, VACV A27 protein, and VACV lysate EIA, we determined the optical density (OD) of individuals aged 0–29 years old, who were born between 1993 and 2022 and therefore have not received smallpox vaccination (*n* = 133) (Fig. 1d). These sera were collected between January and April 2022, before the global mpox outbreak which started in May 2022. By using these cutoffs, we found that the seropositive rates were 2.2% (4/183; [95% confidence interval (CI): 0.9–5.5%]) for MPXV A29 protein, 2.7% (5/183 [95% CI: 1.2–6.2]) for VACV A27 protein, and 2.2% (4/183; [95% CI: 0.9–5.5]) for VACV lysate among individuals aged below 40 years old (Fig. 1e). The VACV lysate seropositive rate started to increase for the age group 40–49 years old (36% [18/50; 95% CI: 24.1-49.9%] and increased to 93.3% [56/60; 95% CI: 84.1-97.4%] for older adults aged 80 years or above (Fig. 1e,  f and Table 1), which coincided with timing of the cessation of the smallpox vaccination program in Hong Kong^19^. In contrast, the MPXV A29 and VACV A27 seropositive rates were 2–6% and 4–12% for all age groups between 40 and 79 years old, respectively; and were 18.3% (11/60] and 26.7% (16/60) for those aged 80 years or above, respectively. MPXV A29 OD was generally higher among serum specimens that were VACV A27 or VACV lysate positive than those that were VACV A27 or VACV lysate negative (Supplementary Fig. S1).

**Table 1:** Comparison of performance of the EIA for MPXV A29 protein, VACV A27 protein, and VACV lysate.

Age group (years)	EIA result			No. (%)
	MPXV A29 protein	VACV A27 protein	VACV lysate	
0–39	+	+	+	0/183 (0)
	+	+	–	1/183 (0.6)
	+	–	+	0/183 (0)
	+	–	–	3/183 (2)
	–	+	+	0/183 (0)
	–	+	–	4/183 (2)
	–	–	+	4/183 (2)
	–	–	–	171/183 (93)
40–49	+	+	+	0/50 (0)
	+	+	–	0/50 (0)
	+	–	+	0/50 (0)
	+	–	–	1/50 (2)
	–	+	+	0/50 (0)
	–	+	–	2/50 (4)
	–	–	+	18/50 (36)
	–	–	–	29/50 (58)
50–59	+	+	+	0/50 (0)
	+	+	–	0/50 (0)
	+	–	+	1/50 (2)
	+	–	–	1/50 (2)
	–	+	+	0/50 (0)
	–	+	–	2/50 (4)
	–	–	+	23/50 (48)
	–	–	–	23/50 (46)
60–69	+	+	+	1/50 (2)
	+	+	–	0/50 (0)
	+	–	+	1/50 (2)
	+	–	-	1/50 (2)
	–	+	+	1/50 (2)
	–	+	–	0/50 (0)
	–	–	+	35/50 (70)
	–	–	–	11/50 (22)
70–79	+	+	+	3/50 (6)
	+	+	–	0/50 (0)
	+	–	+	0/50 (0)
	+	–	–	0/50 (0)
	–	+	+	2/50 (4)
	–	+	–	1/50 (2)
	–	–	+	38/50 (76)
	–	–	–	6/50 (12)
80 or above	+	+	+	6/60 (10)
	+	+	–	0/60 (0)
	+	–	+	5/60 (8.3)
	+	–	–	0/60 (0)
	–	+	+	8/60 (13.3)
	–	+	–	2/60 (3.3)
	–	–	+	37/60 (61.7)
	–	–	–	2/60 (3.3)

**Fig. 2:**
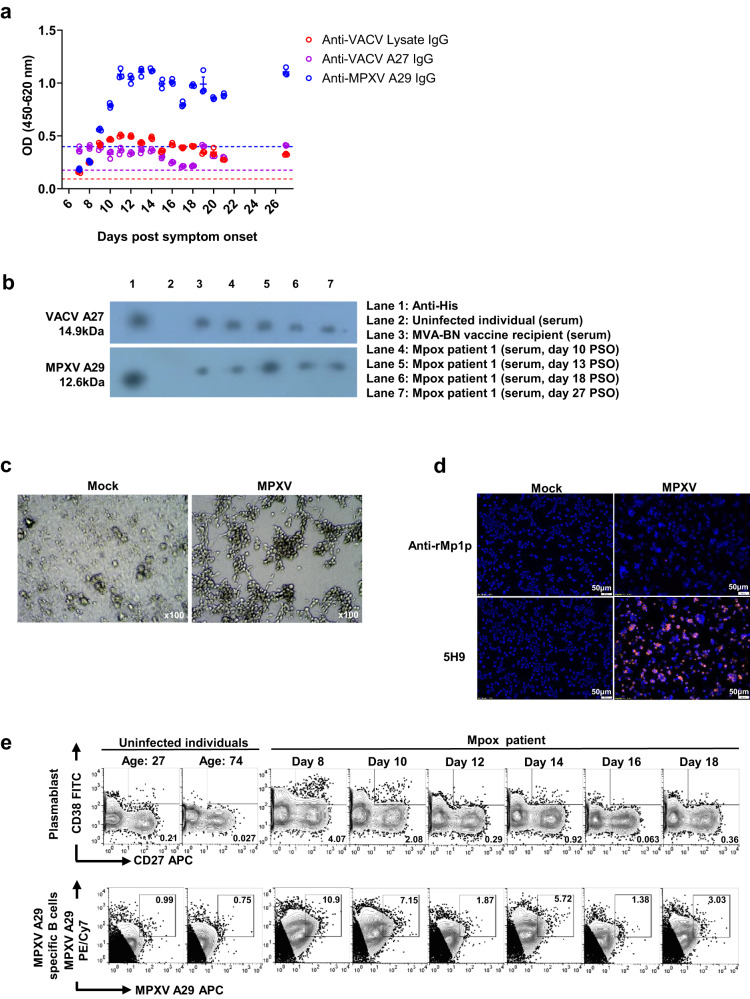
Antibody and B-cell kinetics. **a** MPXV A29 protein (blue), VACV A27 protein (violet), and VACV lysate (red) EIA with serial specimens of mpox patient 1’s serum collected from day 7 PSO till day 21 PSO, and day 27 PSO. The error bar represents the standard derivation of the mean from 3 technical replicates. **b** Western blot for the detection of anti-VACV A27 IgG and anti-MPXV A29 IgG. Specimens included serial serum specimens from patient 1 collected on days 10, 13, 18, and 27 PSO (Lane 4–7), a serum specimen collected from a recipient of the MVA-BN vaccine (collected 8 months after the second dose of vaccine) (Lane 3), and a serum specimen collected from an uninfected individual (within the age group 20–29) (Lane 2, as negative control). Mouse anti-His antibody was used as positive control. The original western blot image is shown in Supplementary Fig. S6b. **c**, **d** MPXV isolated from saliva specimen of mpox patient 1 on day 8 PSO was used to inoculate VeroE6 cells. **c** CPE was observed on day 7 post infection. Uninfected cells were indicated as mock. Magnification x100. **d** VeroE6 cells infected with or without MPXV were detected by mouse anti-rMp1p or mouse anti-MPXV-A29 (5H9) antibodies. The positive signal was examined and analyzed by a fluorescent microscope equipped with Image-Pro Plus 4.5 software. (blue) DAPI, (magenta) MPXV-infected cells. Magnification x126. Scale bar = 50 μm. **e** Peripheral blood mononuclear cells of mpox patient 1 were isolated from whole blood collected on days 8, 10, 12, 14, 16, 18 PSO. The frequency of MPXV A29-specific B cells (CD3^-^CD19^+^ MPXV A29 protein^+^) and plasmablast (CD3^-^CD19^+^CD27^+^CD38^+^) were quantified by flow cytometry. Antigen specificity was determined on the basis of binding to fluorophore-labeled MPXV A29 protein.

In September 2022, we have diagnosed the first symptomatic case in Hong Kong (mpox patient 1 in this study). The patient was a 30-year-old man presenting with infectious mononucleosis-like syndrome, and the clinical details of this patient were reported previously^11^. We collected serial specimens from day 7 post-symptom onset (PSO) (first day of hospital admission) till day 21 PSO (day of hospital discharge). Subsequent specimens on day 27 PSO were collected during follow-up at out-patient clinic. Anti-MPXV A29 IgG became positive (above the cutoff: 0.398) on day 9 PSO and peaked on day 11 PSO (Fig. 2a). The presence of anti-MPXV A29 IgG in the mpox patient 1’s sera was confirmed using western blot (Fig. 2b).

Furthermore, we have collected the convalescent serum (collected 6 days PSO) from another mpox patient (mpox patient 2 in this study), who was a 47-year-old man who presented to our hospital in August 2023 with a penile lesion. Mpox patient 2 received two doses of the MPV-BN vaccine previously. We have also collected a serum specimen from a vaccine recipient 8 months after the second dose of the MVA–BN vaccine (MVA-BN vaccine recipient). Both mpox patient 2 and the MVA-BN vaccine recipient tested positive for anti-VACV lysate IgG and anti-VACV A27 IgG (Supplementary Fig. S2a). Mpox patient 2 also tested positive for anti-MPXV A29 IgG. For the MVA-BA vaccine recipient, anti-MPXV A29 IgG could be detected by western blot (Fig. 2b), but the OD was slightly below the cutoff in the EIA (Supplementary Fig. S2a). The MPXV A29 IgG OD was higher than the VACV A27 IgG OD for both mpox patients (2.75-fold and 1.84-fold for mpox patient 1 and mpox patient 2, respectively) (Supplementary Fig. S2b). However, the MPXV A29 IgG OD was slightly lower than the VACV A27 IgG OD for the MVA-BN vaccine recipient.

We performed a viral culture from the saliva specimen of this patient using the VeroE6 cell line. CPE was seen from the saliva specimens collected from day 8 to day 11 PSO and a representative image of day 8 PSO is shown in Fig. 2c. Infection of the VeroE6 cell line was confirmed by immunofluorescence staining of the MPXV A29 protein using the anti-MPXV A29 antibody 5H9 (Fig. 2d).

Next, we tested if MPXV A29 protein can be used to identify MPXV A29-specific B cells in mpox patients. We first assessed the kinetic of plasmablast (CD3^-^CD19^+^CD27^+^CD38^+^), the antibody-secreting cell response of mpox patient 1. The percentage of plasmablast (4.07% of CD3^-^CD19^+^ B cells) peaked on day 8 PSO and gradually decreased (Fig. 2e). This is correlating to the anti-MPXV A29 antibody level that initiated rising on day 9 PSO (Fig. 2a). We next assessed the kinetic of MPXV A29-specific B cell. As observed with the plasmablast kinetic, the frequency of MPXV A29-specific B cells (10.9% of CD3^-^CD19^+^ B cells) also peaked on day 8 PSO and gradually decreased (Fig. 2e and Supplementary Fig. S3). Among the plasmablast population detected, 0.81% was identified as MPXL A29-specific (Supplementary Fig. S4).

## Discussion

Our findings indicate that MPXV A29 protein can be used for antibody and B-cell assays, and anti-MPXV A29 antibody can be used for immunofluorescence assay. Unlike VACV or MPXV lysate, the recombinant MPXV A29 protein can be produced in large quantity without viral culture facilities, which eliminate the potential biohazards associated with live MPXV. Recombinant protein would have less batch-to-batch variations than MPXV cell lysate. Furthermore, serial testing of serum specimens from mpox patient 1 showed that there was an increase in the levels of antibodies against the MPXV A29 protein but not the VACV A27 protein. These advantages make MPXV A29 protein a useful target for vaccine studies in monitoring antibody and B-cell response.

The seropositive rate of MPXV A29 protein EIA was 2–6% for age groups between 40 and 79 years old, which was much lower than the seropositive rate of VACV lysate EIA for the same age groups (36%–86%). Our results indicate that MPXV A29 protein was less affected by prior smallpox vaccine or prior smallpox infection than VACV lysate, and therefore a preferred assay for MPXV serosurveillance studies. However, the seropositive rates of VACV A27 protein were only slightly higher than those of MPXV A29 protein (Fig. 1e). Therefore, the differences between VACV lysate and MPXV A29 protein were most likely related to antibodies binding to non-A27/A29 targets. Interestingly, the seropositive rate of VACV lysate EIA increased with older age. One possibility is that these older individuals may have had natural infection previously, and that antibody waning may be slower among individuals who were exposed to the smallpox virus.

Although the mouse monoclonal antibody against MPXV (5H9) cross-reacted with VACV A27 protein, the convalescent serum of mpox patient 1 had minimal cross-reactivity with VACV A27 protein. Our results suggest that the polyclonal antibodies generated after natural infection in the patient may specifically target the epitopes that are different between MPXV A29 protein and VACV A27 protein. However, since we only tested the serum of two mpox patients and one MVA-BN vaccine recipient, it remains to be determined whether these results could be generalized. It would also be important to determine how immunocompromised conditions would affect the antibody response.

Mpox patients had high levels of viral shedding in saliva. Hernaez et al. found that MPXV could be detected in the saliva by PCR in 85% of patients, while viral culture was positive in 67% of PCR-positive saliva samples^20^. In our current study, we were able to isolate live virus from our patient’s saliva specimens up to day 11 PSO. Our results suggest the possibility of prolonged transmission of MPXV via saliva either directly or indirectly.

There are several limitations in this study. First, as we only have two laboratory-confirmed mpox patients and one MVA-BN vaccine recipient, we were not able to determine the sensitivity of our MPXVA29 protein EIA. Second, our mpox patient 1 presented with infectious mononucleosis-like syndrome, which was not commonly reported. Hence, it is unclear whether the B cell and plasmablast response in this patient is also seen in other patients without infectious mononucleosis-like syndrome.

In summary, MPXV A29 is a suitable protein for studying antibody and B-cell response among mpox patients. While the ortholog VACV A27 protein was shown to be a neutralizing antibody epitope, further studies are required to determine whether the level of antibody against MPXV A29 protein correlates with protection.

### Supplementary information

Supplementary Information

Description of Additional Supplementary Files

Supplementary Data

Reporting Summary

## Data Availability

The source data underlying Table 1, Figs. 1c, d, f, and 2a are available in Supplementary Data 1.
