# Supplementary material for: Determination of seroprevalence and kinetics of humoral response using mpox virus A29 protein
Source: Commun Med (Lond). 2023 Nov 22;3:168. doi: 10.1038/s43856-023-00403-9 (PMC10665351; doi:10.1038/s43856-023-00403-9)
Supplement: Supplementary file 1 — Supplementary Information [file 43856_2023_403_MOESM1_ESM.pdf]

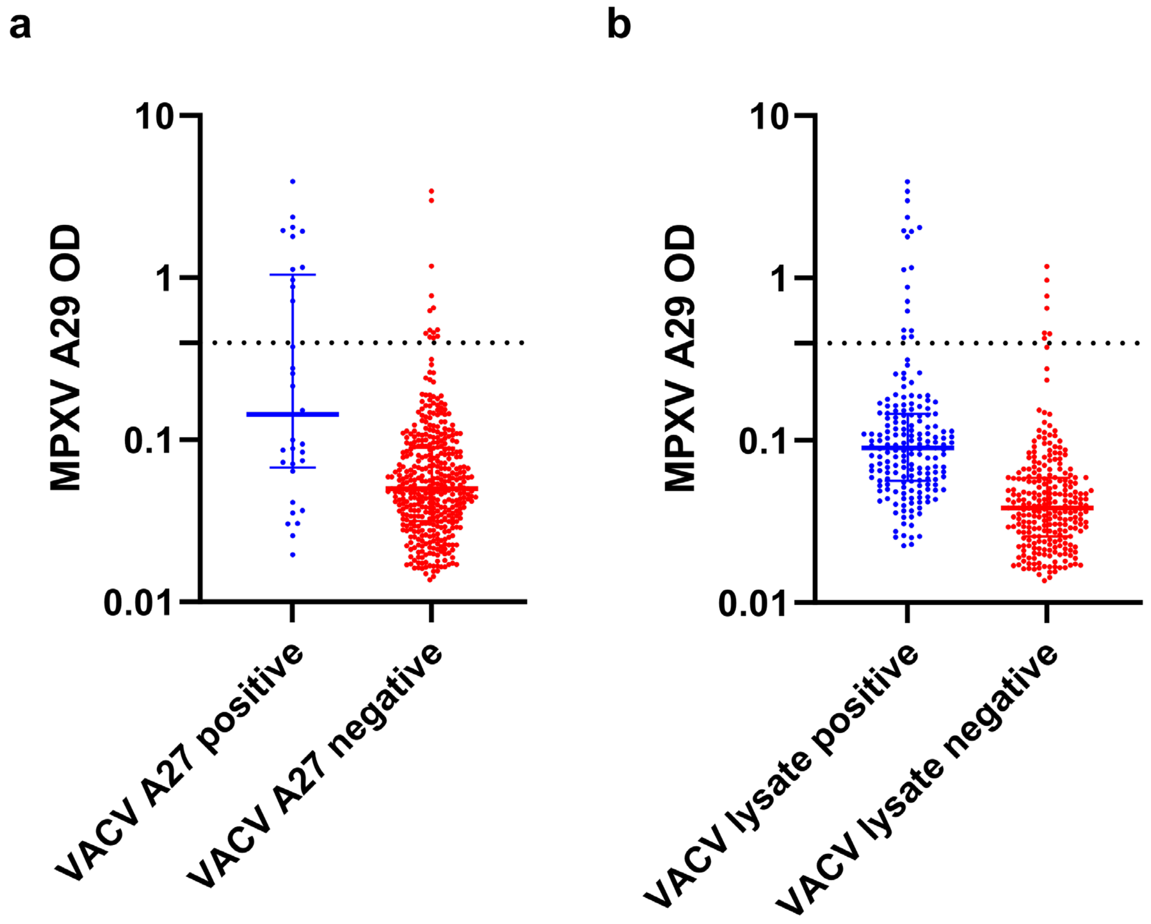

Supplementary Fig. S1. Comparison of the anti-MPXV A29 IgG OD values between a) VACV A27 positive and VACV A27 negative sera, and between b) VACV lysate positive and VACV lysate negative sera.

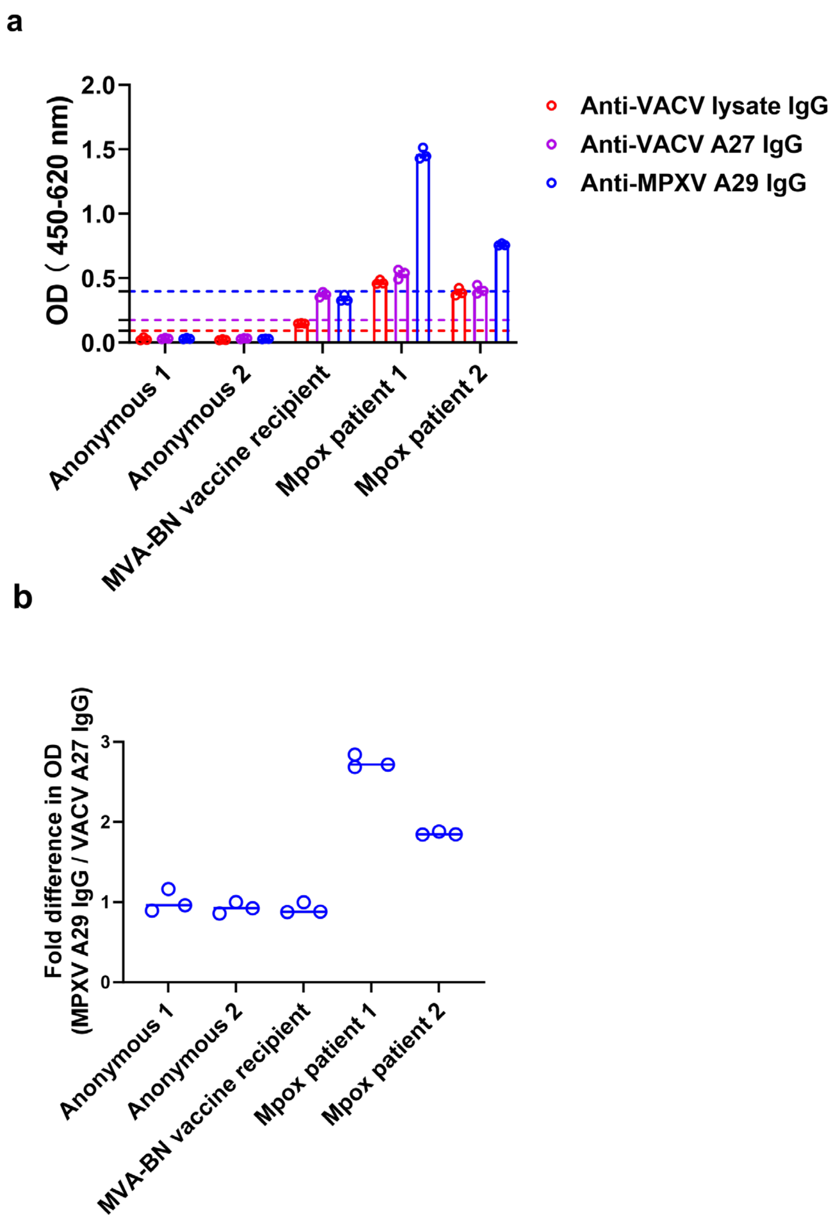

Supplementary Fig. S2. a) Comparison of anti-VACV lysate IgG, anti-VACV A27 IgG and anti-MPXV A29 IgG between non-infected non-vaccinated individuals (anonymous 1 [age group 20-29 years] and anonymous 2 [age group 30-39 years]), the MVA-BN vaccine recipient [age group 20-29 years; serum collected 8 months after second dose of MVA-BN vaccine]), mpox patient 1 (serum collected 27 days PSO) and mpox patient 2 (serum collected 6 days PSO). b) Comparison between the fold difference in the OD between MPXV A29 IgG and VACV A27 IgG.

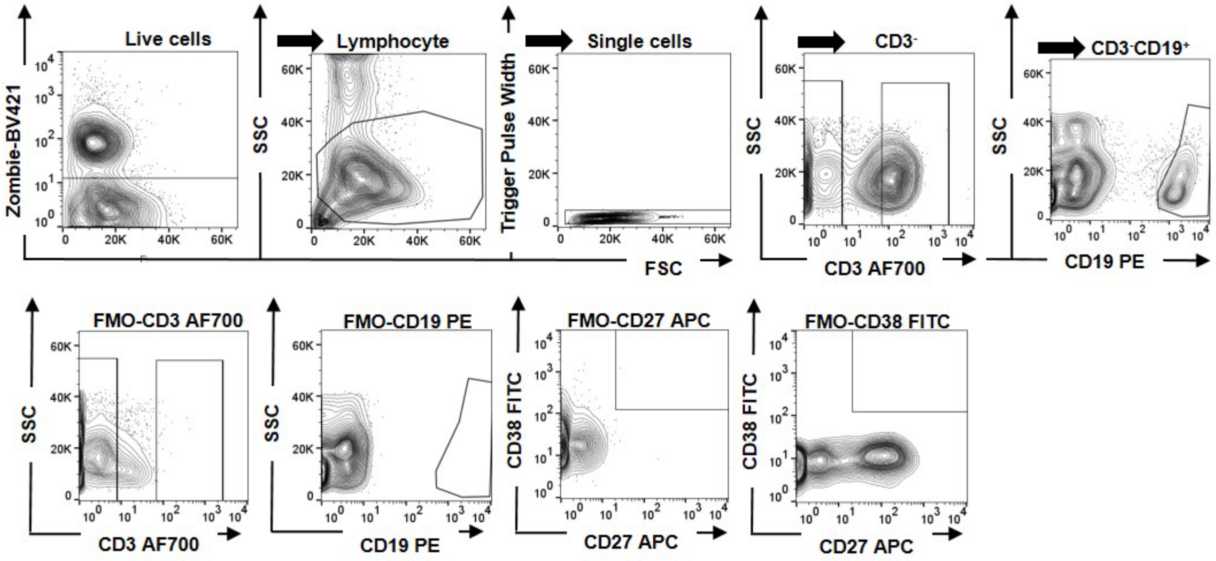

Supplementary Fig. S3. Flow cytometry gating strategy for plasmablast and MPXV A29 specific B cells for figure 2e.

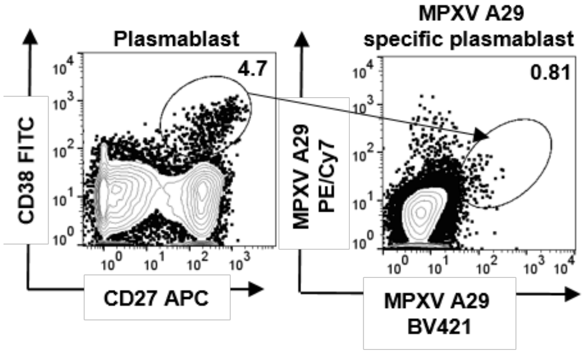

Supplementary Fig. S4. PBMC of mpox patient were collected on day 9 PSO. The percentage of MPXV A29 specific plasmablast (CD3-CD19+CD27+CD38+) population.

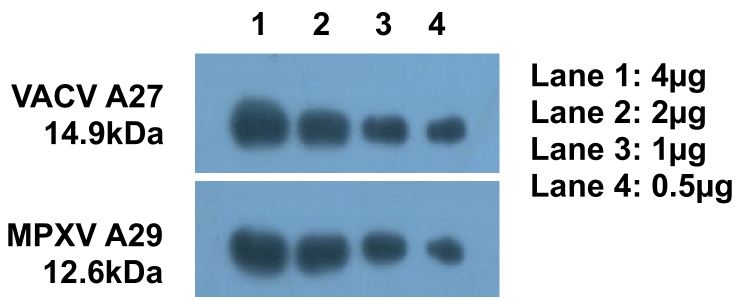

Supplementary Fig. S5. Comparison of the expression of VACV A27 and MPXV A29 recombinant proteins by Western blot.

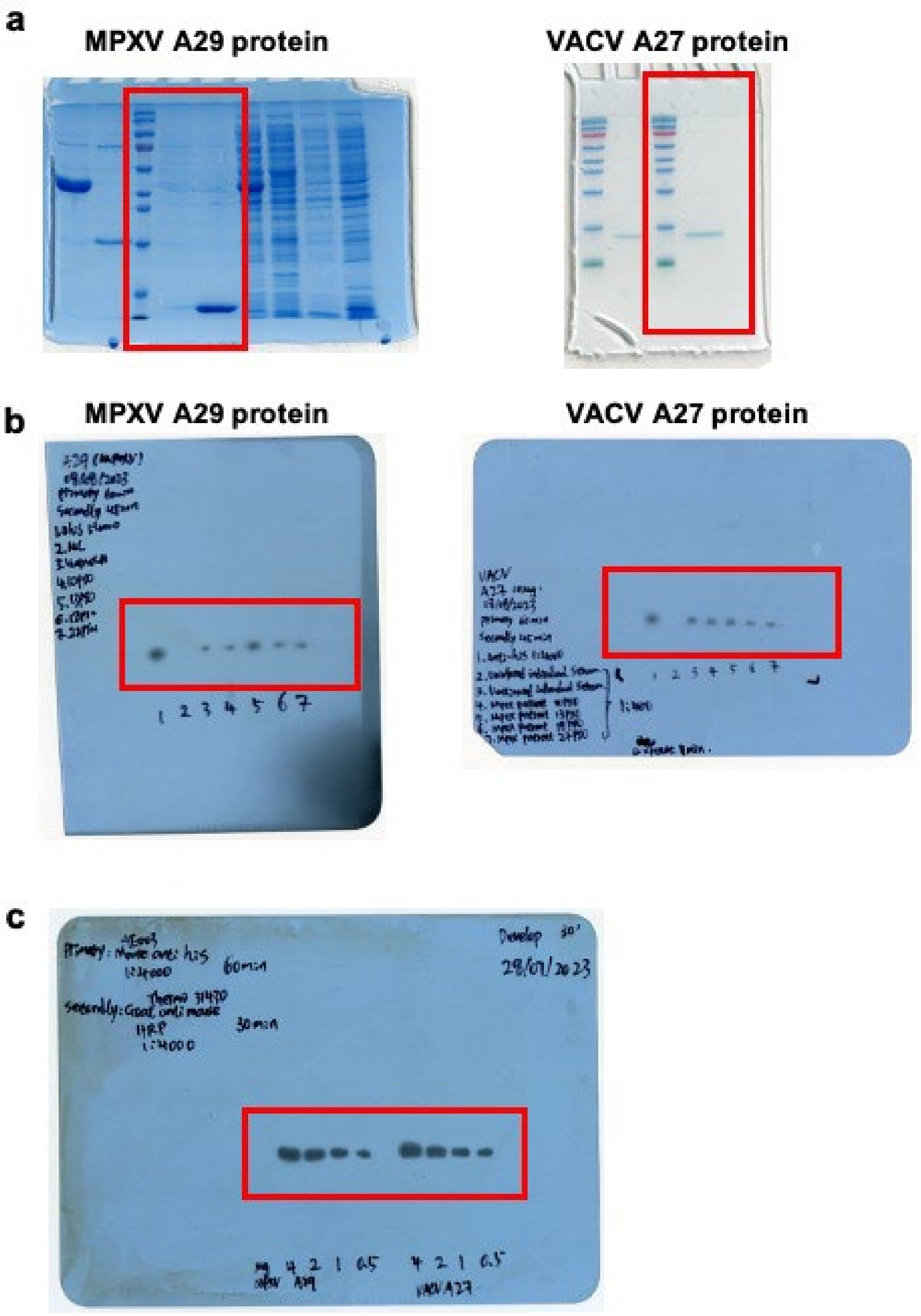

Supplementary Fig. S6. Original images for a) coomassie blue stained SDS-PAGE in Fig. 1b; b) Western blot in Fig. 2b. c) Western blot in Supplementary Fig. S5.
