# Supplementary material for: Determination of seroprevalence and kinetics of humoral response using mpox virus A29 protein
Source: Commun Med (Lond). 2023 Nov 22;3:168. doi: 10.1038/s43856-023-00403-9 (PMC10665351; doi:10.1038/s43856-023-00403-9)
Supplement: Supplementary file 2 — Description of Additional Supplementary Files [file 43856_2023_403_MOESM2_ESM.pdf]

# Description of Additional Supplementary Files

**File Name:** Supplementary Data
**Description:** Numerical Data for Table 1, Figure 1c, Figure 1d, Figure 1f and Figure 2a.
