# Supplementary material for: Determination of seroprevalence and kinetics of humoral response using mpox virus A29 protein
Source: Commun Med (Lond). 2023 Nov 22;3:168. doi: 10.1038/s43856-023-00403-9 (PMC10665351; doi:10.1038/s43856-023-00403-9)
Supplement: Supplementary file 4 — Reporting Summary [file 43856_2023_403_MOESM4_ESM.pdf]

nature portfolio

Corresponding author(s): COMMSMED-23-0003-B

Last updated by author(s): Sep 26, 2023

Reporting Summary

Nature Portfolio wishes to improve the reproducibility of the work that we publish. This form provides structure for consistency and transparency in reporting. For further information on Nature Portfolio policies, see our Editorial Policies and the Editorial Policy Checklist.

Statistics

For all statistical analyses, confirm that the following items are present in the figure legend, table legend, main text, or Methods section.

n/a	Confirmed	
☐	☒	The exact sample size (*n*) for each experimental group/condition, given as a discrete number and unit of measurement
☐	☒	A statement on whether measurements were taken from distinct samples or whether the same sample was measured repeatedly
☐	☒	The statistical test(s) used AND whether they are one- or two-sided<br>*Only common tests should be described solely by name; describe more complex techniques in the Methods section.*
☒	☐	A description of all covariates tested
☒	☐	A description of any assumptions or corrections, such as tests of normality and adjustment for multiple comparisons
☐	☒	A full description of the statistical parameters including central tendency (e.g. means) or other basic estimates (e.g. regression coefficient) AND variation (e.g. standard deviation) or associated estimates of uncertainty (e.g. confidence intervals)
☒	☐	For null hypothesis testing, the test statistic (e.g. *F*, *t*, *r*) with confidence intervals, effect sizes, degrees of freedom and *P* value noted<br>*Give P values as exact values whenever suitable.*
☒	☐	For Bayesian analysis, information on the choice of priors and Markov chain Monte Carlo settings
☒	☐	For hierarchical and complex designs, identification of the appropriate level for tests and full reporting of outcomes
☒	☐	Estimates of effect sizes (e.g. Cohen's *d*, Pearson's *r*), indicating how they were calculated

Our web collection on statistics for biologists contains articles on many of the points above.

Software and code

Policy information about availability of computer code

Data collection	Protein sequence were aligned with PRALINE. ELISA data were collected by Thermoscientific Multiskan. Immunofluorescence images were obtained by Olympus BX53 microscope. Flow cytometry data were collected by BD influx
Data analysis	EIA data were analysed by SkanIt Software 6.0.2 for Microplate Readers RE, (verion 6.0.2.3). Immunofluorescence images were analysed by Olympus cellSens software (version 1.17). Flow cytometry data were analysed by Flowjo (version 10.5.2). GraphPad Prism (Version 9.1.2.) was used for statistic analysis.

For manuscripts utilizing custom algorithms or software that are central to the research but not yet described in published literature, software must be made available to editors and reviewers. We strongly encourage code deposition in a community repository (e.g. GitHub). See the Nature Portfolio guidelines for submitting code & software for further information.

Data

Policy information about availability of data

All manuscripts must include a data availability statement. This statement should provide the following information, where applicable:

- Accession codes, unique identifiers, or web links for publicly available datasets
- A description of any restrictions on data availability
- For clinical datasets or third party data, please ensure that the statement adheres to our policy

Accession number YP_233032.1 - https://www.ncbi.nlm.nih.gov/protein/YP_233032.1

Accession number AAL40597.1 - https://www.ncbi.nlm.nih.gov/protein/AAL40597.1
Accession number NP 042178.1 - https://www.ncbi.nlm.nih.gov/search/all/?term=NP%20042178.1
Monkeypox virus strain Zaire-96-I-16, complete genome - https://www.ncbi.nlm.nih.gov/nuccore/AF380138.1
Vaccinia virus, complete genome - https://www.ncbi.nlm.nih.gov/nuccore/NC_006998.1
Vaccinia virus (strain Tian Tan) complete genome - https://www.ncbi.nlm.nih.gov/nuccore/AF095689.1

Data relating to the findings of this study are available from the corresponding author upon request.

Human research participants

Policy information about studies involving human research participants and Sex and Gender in Research.

Reporting on sex and gender	A total of 443 serum specimens collected between January and April 2022 were randomly selected and tested, without knowing the gender.
Population characteristics	The archived specimens encompassed all age groups from 0-9 to ≥80 years old. A total of 443 serum specimens collected between January and April 2022 were randomly selected and tested.
Recruitment	Anonymised archived serum samples from the clinical biochemistry laboratory of Queen Mary Hospital in Hong Kong. Written informed consent was waived since archived anonymized specimens were used.
Ethics oversight	This study was approved by the Institutional Review Board of the University of Hong Kong/Hospital Authority Hong Kong West Cluster (UW 13-265 and UW 18-141).

Note that full information on the approval of the study protocol must also be provided in the manuscript.

Field-specific reporting

Please select the one below that is the best fit for your research. If you are not sure, read the appropriate sections before making your selection.

☒ Life sciences ☐ Behavioural & social sciences ☐ Ecological, evolutionary & environmental sciences

For a reference copy of the document with all sections, see nature.com/documents/nr-reporting-summary-flat.pdf

Life sciences study design

All studies must disclose on these points even when the disclosure is negative.

Sample size	The sample size of the anonymized sera was based on the availability. No sample size calculation was performed.
Data exclusions	All samples with available data were included in the respective analyses.
Replication	Archived serum specimen from different individuals of the same age group was used to replicate findings.<br>Experiments did not include replicates as all participants and data points are unique.<br>Experiment used technical replicates were stated in the manuscript accordingly.<br>Some results were confirmed by different methods.
Randomization	Without knowing the gender and medical history, anonymised archived serum samples of each age group were randomly selected from clinical biochemistry laboratory of Queen Mary Hospital in Hong Kong. These samples were used for serological study in our previously published data on SARS-CoV-2 (To, K. K. et al. The Lancet Microbe 2020).
Blinding	Blinding was not appropriate for this study with healthy individual and monkeypox virus infected patient.

Reporting for specific materials, systems and methods

We require information from authors about some types of materials, experimental systems and methods used in many studies. Here, indicate whether each material, system or method listed is relevant to your study. If you are not sure if a list item applies to your research, read the appropriate section before selecting a response.

Materials & experimental systems

n/a	Involved in the study
☐	☒ Antibodies
☐	☒ Eukaryotic cell lines
☒	☐ Palaeontology and archaeology
☒	☐ Animals and other organisms
☐	☒ Clinical data
☒	☐ Dual use research of concern

Methods

n/a	Involved in the study
☒	☐ ChIP-seq
☐	☒ Flow cytometry
☒	☐ MRI-based neuroimaging

Antibodies

Antibodies used

1) Alexa Fluor 700 anti-human CD3, clone SK7. BioLegend, Cat# 344822, AB_2563420
2) Alexa Fluor 700 Mouse IgG1 Isotype Ctrl, clone MOPC-21, BioLegend, Cat# 400144
3) PE anti-human CD19, clone: HIB19, BioLegend, Cat#302208, AB_314238
4) PE Mouse IgG1 k Isotype Ctrl, clone MOPC -21, BioLegend, Cat#40011
5) APC anti-human CD27, clone: O323, BioLegend, Cat#302810, AB_314302
6) APC Mouse IgG1 k Isotype Ctrl, clone MOP-21, Cat# 400120
7) FITC anti-human CD38, clone HB-7, Cat#356610, Lot#B375900, AB_2561950
8) FITC Mouse IgG1 k Isotype Ctrl, clone MOPC- 21, Cat#400110

9) Alexa Fluor 594 - anti-mouse IgG, Thermofisher Scientific, Cat# A11005, RRID:AB_2534073
10) Goat anti-human IgG HRP, Thermofisher Scientific, Cat# A18811, RRID:AB_2535588
11) Goat anti-mouse IgG HRP, Thermofisher Scientific, Cat# 31430, RRID:AB_228307
12) Mouse anti-HisTag antibody, ABclonal, Cat# AE003, RRID:AB_2728734
13) In house: mouse anti-MPXV A29L (5H9)
14) In house: mouse anti-rMp1p

Validation

All antibodies applied in current study are validated by the manufacturer for their species specificity and application. Validation statements from the manufacturer are as follow:

1) Alexa Fluor 700 anti-human CD3, clone SK7. BioLegend, Cat# 344822, AB_2563420
https://www.biolegend.com/en-us/products/alexa-fluor-700-anti-human-cd3-antibody-9738

2) Alexa Fluor 700 Mouse IgG1 Isotype Ctrl, clone MOPC-21, BioLegend, Cat# 400144
https://www.biolegend.com/en-us/products/alexa-fluor-700-mouse-igg1-kappa-isotype-ctrl-3376

3) PE anti-human CD19, clone: HIB19, BioLegend, Cat#302208, AB_314238
https://www.biolegend.com/en-us/products/pe-anti-human-cd19-antibody-719

4) PE Mouse IgG1 k Isotype Ctrl, clone MOPC -21, BioLegend, Cat#400112
https://www.biolegend.com/en-us/products/pe-mouse-igg1-kappa-isotype-ctrl-1408

5) APC anti-human CD27, clone: O323, BioLegend, Cat#302810, AB_314302
https://www.biolegend.com/en-us/products/apc-anti-human-cd27-antibody-808

6) APC Mouse IgG1 k Isotype Ctrl, clone MOP-21, Cat# 400120
https://www.biolegend.com/en-us/products/apc-mouse-igg1-kappa-isotype-ctrl-1404

7) FITC anti-human CD38, clone HB-7, Cat#356610, Lot#B375900, AB_2561950
https://www.biolegend.com/en-us/products/fitc-anti-human-cd38-antibody-8468

8) FITC Mouse IgG1 k Isotype Ctrl, clone MOPC- 21, Cat#400110
https://www.biolegend.com/en-us/products/fitc-mouse-igg1-kappa-isotype-ctrl-fc-3036

9) Alexa Fluor 594 - anti-mouse IgG, Thermofisher Scientific, Cat#A11005, RRID:AB_2534073
https://www.thermofisher.com/antibody/product/A-11005.html?gclid=CjwKCAjw9pGjBhB-EiwAa5jl3GWRDTRIUhIrn1fjJzatL7dFebOlyWdCnj9UAYv6HOvAl1LSpQbHVRoCIQsQAvD_BwE&ef_id=CjwKCAjw9pGjBhB-EiwAa5jl3GWRDTRIUhIrn1fjJzatL7dFebOlyWdCnj9UAYv6HOvAl1LSpQbHVRoCIQsQAvD_BwE:G:s&s_kwcid=AL!3652!3!516608152455!!!g!!!12825517856!122158234995&cid=bid_pca_aus_r01_co_cp1359_pjt0000_bid00000_0se_gaw_dy_pur_con

10) Goat anti-human IgG HRP, Thermofisher Scientific, Cat#A18811, RRID:AB_2535588
https://www.thermofisher.com/antibody/product/Goat-anti-Human-IgG-H-L-Cross-Adsorbed-Secondary-Antibody-Polyclonal/A18811

11) Goat anti-mouse IgG HRP, Thermofisher Scientific, Cat#31430, RRID:AB_228307
https://www.thermofisher.com/antibody/product/Goat-anti-Mouse-IgG-H-L-Secondary-Antibody-Polyclonal/31430

12) Mouse anti-HisTag antibody, ABclonal, Cat#AE003, RRID:AB_2728734
In house: mouse anti-MPXV A29L (5H9)

13) Mouse anti-MPXV A29L (5H9) produced in house was validated in current study.

14) In house: mouse anti-rMp1p
Mouse anti-Mp1p antibody produced in house was validated and published in PLoS One. 2011;6(12):e28796.

Eukaryotic cell lines

Policy information about cell lines and Sex and Gender in Research

Cell line source(s)	BL21-Gold (DE3), Agilent, Cat#230132<br>VeroE6 cells, ATCC, Cat#CRL-15786<br>NS1 (myeloma cells), ATCC, Cat#TIB-18TM
Authentication	Cell lines were not authenticated
Mycoplasma contamination	We verified the cells free of mycoplasma contamination by using the mycoplasma screening service provided by HKU core facilities.
Commonly misidentified lines (See ICLAC register)	No commonly misidentified lines were used.

Clinical data

Policy information about clinical studies
All manuscripts should comply with the ICMJE guidelines for publication of clinical research and a completed CONSORT checklist must be included with all submissions.

Clinical trial registration	This is not a clinical trial
Study protocol	This is not a clinical trial
Data collection	Not applicable.
Outcomes	Not applicable

Flow Cytometry

Plots

Confirm that:

☒ The axis labels state the marker and fluorochrome used (e.g. CD4-FITC).

☒ The axis scales are clearly visible. Include numbers along axes only for bottom left plot of group (a 'group' is an analysis of identical markers).

☒ All plots are contour plots with outliers or pseudocolor plots.

☒ A numerical value for number of cells or percentage (with statistics) is provided.

Methodology

Sample preparation	Cryopreserved PBMCs were first blocked with human FcR blocking reagent and dead cells were stained with Zombie NIR/Zombie violet Fixable Viability Kit. PBMC were then stained with MPXV A29L BV421/APC and MPXV A29L PE/Cy7 for 1 h at 4oC and followed by surface antibody staining with Alexa Fluor 700 anti-human CD3, PE anti-human CD19, APC anti-human CD27, FITC anti-human CD38 antibodies.
Instrument	Data were analysed using BD influx (BD).
Software	Data were analysed using flowjo, version 10.5.2 (BD).
Cell population abundance	Cells were not enriched or sorted prior FACS staining.
Gating strategy	Viable cells were determined using Zombie NIR/Zombie violet. Lymphocyte was gated from PBMC with the FSC/SSC gate. Single cells were selected. Live cells were gated for CD3 negative and CD19 positive cells.

☒ Tick this box to confirm that a figure exemplifying the gating strategy is provided in the Supplementary Information.
